# Long- and Short-Term Health Effects of Pesticide Exposure: A Cohort Study from China

**DOI:** 10.1371/journal.pone.0128766

**Published:** 2015-06-04

**Authors:** Ruifa Hu, Xusheng Huang, Jikun Huang, Yifan Li, Chao Zhang, Yanhong Yin, Zhaohui Chen, Yanhong Jin, Jinyang Cai, Fang Cui

**Affiliations:** 1 School of Management and Economics, Beijing Institute of Technology, Beijing, China; 2 Department of Neurology, Chinese PLA General Hospital, Beijing, China; 3 Center for Chinese Agricultural Policy, Institute of Geographic Sciences and Natural Resources Research, Chinese Academy of Sciences, Beijing, China; 4 Department of Agricultural, Food and Resource Economics, Rutgers University, New Brunswick, New Jersey, United States of America; Hospital General Dr. Manuel Gea González, MEXICO

## Abstract

Pesticides are extensively used by farmers in China. However, the effects of pesticides on farmers’ health have not yet been systematically studied. This study evaluated the effects of pesticides exposure on hematological and neurological indicators over 3 years and 10 days respectively. A cohort of 246 farmers was randomly selected from 3 provinces (Guangdong, Jiangxi, and Hebei) in China. Two rounds of health investigations, including blood tests and neurological examinations, were conducted by medical doctors before and after the crop season in 2012. The data on pesticide use in 2009–2011 were collected retrospectively via face-to-face interviews and the 2012 data were collected from personal records maintained by participants prospectively. Ordinary least square (OLS), Probit, and fixed effect models were used to evaluate the relationship between pesticides exposure frequency and the health indicators. Long-term pesticide exposure was found to be associated with increased abnormality of nerve conductions, especially in sensory nerves. It also affected a wide spectrum of health indicators based on blood tests and decreased the tibial nerve compound muscle action potential amplitudes. Short-term health effects included alterations in complete blood count, hepatic and renal functions, and nerve conduction velocities and amplitudes. However, these effects could not be detected after 3 days following pesticide exposure. Overall, our results demonstrate that pesticide exposure adversely affects blood cells, the liver, and the peripheral nervous system. Future studies are needed to elucidate the specific effects of each pesticide and the mechanisms of these effects.

## Introduction

Pesticide use in crop production worldwide increased nearly twentyfold from 1960 to 2000 [1] and further increased from 1.0 billion tons in 2002 to 1.7 billion tons in 2007 [2]. China is the largest producer of pesticides and one of the most intensive pesticide users in crop production in the world [3, 4]. Despite the well-documented deleterious effects of pesticides on biological pest control function, the environment, and food safety [5–7], the health effects of these agents have also attracted substantial attention [8].

Previous studies showed that pesticide exposure often induces acute and chronic neurological toxicity [9–14] and dysfunctional lipid, protein, and carbohydrate metabolism [15]. However, most surveys used subjective or qualitative measurements, such as symptoms, psychological scales, or clinical signs, to evaluate the health effects of pesticide use [16–18]. For example, one study found that farmers who used a greater amount of pesticides were more likely to suffer from headache, nausea, and skin problems [13]. These outcomes had limitations in the following two aspects. First, the reported symptoms may not be necessarily pathological. Second, there may be subclinical neuroelectrophysiological changes when no clinical symptoms or signs were found. Our study relies on objective and quantitative measures of neuroelectrophysiology to evaluate health effects of pesticide exposure. Furthermore, the relationship between pesticide exposure and human health remains unclear because the time and extent of pesticide exposure were not sufficiently estimated in many previous studies [19]. The majority of studies used one-time household survey data to examine the health effects of pesticide exposure [9, 13, 20], and used nonspraying workers [21] or the general population [16] as non-exposed controls. While these studies did provide evidence of the adverse health effects of pesticide exposure, it was difficult to effectively control for the potentially confounding health-related factors of each participant and to accurately define exposure level. The objective of the present study is to use the most detailed neuroelectrophysiological methods to examine the clinical and subclinical health effects of pesticide exposures based on a unique dataset that covers 2 time periods in China.

## Materials and Methods

### Sample selection

This study was conducted in Guangdong, Jiangxi, and Hebei provinces, which represent high, middle, and low levels of pesticide applications in China, respectively. In each province, 2 counties and 2 villages from each county were randomly selected. In each village, 20–25 households were randomly sampled. In total, 246 households participated in this study. The samples differ slightly in terms of health examinations because a few participants were absent during health investigations (S1 Table).

### Exposure estimation

Pesticide exposure information was obtained from 2 datasets. We first collected baseline data regarding pesticide use history, household and individual characteristics, and pesticide-related poisoning events via face-to-face interviews. Participants were asked to report the frequency of pesticide application, and whether they had suffered from headache, nausea, skin irritation, or digestive discomfort that was severe enough to interfere with their work during the past three years (2009–2011). These data were used to assess the long-term health effects of pesticide exposure. All participants were divided into 2 groups. One group had been exposed to relatively high pesticide levels (more than 50 times of pesticide application in 2009–2011, Group H), and the other group had been exposed to lower pesticide levels (less than 50 times of pesticide application in 2009–2011, Group L). Secondly, participants were asked to record each pesticide application in 2012 on the specially designed calendar we had provided them. Moreover, this recording process was supervised by the village leaders under the guidance of the research team. We checked the record at least once a month to ensure its validity and reliability. We used the frequency of pesticide application in the previous 3 days and 4–10 days to analyze short-term effect of pesticide application.

### Health investigations

Two rounds of health investigation were conducted. The first was in March 2012, before participants had applied pesticides for their crop production. The second one was during the course of crop production but prior to crop harvesting. In total, 66 health indicators were examined during health investigations. The list of measured health indicators is shown in S1 Table. Definitions of all measured health variables are provided in S2 and S3 Tables. For each health indicator, abnormality was defined as the corresponding value falls out of the normal range.

#### Blood tests

All blood samples were obtained after participants had fasted for 12 hours. Samples were centrifuged immediately and transported in a refrigerated state to the same laboratory in Beijing within 8 hours. The tests included a complete blood count (CBC), blood chemistry panel (e.g., liver function, cholinesterase, renal function, electrolytes, vitamin B_12_, folic acid, fasting plasma glucose) and measurement of C-reactive protein.

#### Clinical examinations

The clinical examinations consisted of general and neurological examinations. The former comprised height, weight, and blood pressure measurements. The latter consisted of the clinical total neuropathy score (TNSc) and the mini-mental state examination (MMSE). The TNSc was administered to all subjects by two certified neurologists who were trained to ensure the results were comparable. The TNSc is composed of seven items: sensory symptoms, motor symptoms, autonomic symptoms, pin sensibility, vibration sensibility, muscle strength, and deep tendon reflexes [22]. For each item, the score ranged between 0 (normal) and 4 (worst), so that the total score range was 0–21. Participants who received a score of 2 or greater were judged to be abnormal.

#### Nerve conduction studies

The conventional nerve conduction studies were performed on four motor (median, ulnar, tibial, and peroneal) and three sensory (median, ulnar, and sural) nerves on the participant’s non-dominant side using surface electrodes with standard placement. The parameters included motor conduction velocity (MCV), distal motor latency (DML), sensory conduction velocity, compound muscle action potential (CMAP), and sensory nerve action potential (SNAP) amplitudes. Participants’ limbs were heated: the lower limb was maintained at 30–33°C, and the upper limb was maintained at 32–34°C during the evaluation. The normal reference ranges were developed at the Neuroelectrophysiology Unit, Department of Neurology, Chinese People’s Liberation Army General Hospital (S1 Table). The study was approved by the Ethics Committee of Chinese PLA General Hospital and all the participants gave their informed, written consent.

### Statistical methods

To control for the impact of confounding factors, multivariate regression analysis was performed. The model used to evaluate the long-term effects of pesticide use on farmers’ health is as follows:

$$Indicator_{i}=a_{0}+a_{1}*Pesticide_{it}+a_{2}*Characteristic_{it}+a_{3}*\text{Re}\;gion_{it}+e_{it}$$ (1)

where *Indicator*_*i*_ indicates an health indictor for farmer *i*. The independent variable of interest, *pesticide*, takes value of 1 if farmer i is in Group H and zero for Group L. *Characteristic* is a vector of the following farmer characteristics: gender (*female = 1; male = 0*), age (*year)*, education (*year*), height (*cm*), weight (*kg*), current smoker (*smoker = 1*, *non-smoker = 0*), and current drinker (*drinker = 1*, *non-drinker = 0*). Finally, regional dummies, Guangdong and Jiangxi, were added into the equation. When, Eq (1) is estimated by the OLS model when *indicator* is a continuous variable. Otherwise, it is an ordered probit model (Dprobit) if *indicator* is a dichotomous variable.

The health indicators from the 2 rounds of health investigations comprise panel data, which allowed us to estimate the short-term effects of pesticide use on farmers’ health, using the farmer’s fixed effect model

$$\Delta Indicator_{i}=\beta_{0}+\beta_{1}*\Delta Pesticide_{i}+\varepsilon_{i}$$ (2)

where ∆*Indicator*_*i*_ is the change in the *i* ^*th*^ farmer’s health indicator during the two rounds of health investigations and ∆*Pesticide*_*i*_ represents additional pesticide use over the same period. All analyses were conducted with STATA 11.0 software. Statistical tests were two-sided, and p-values of < 0.05 were considered statistically significant.

## Results

### Overview of crop pesticide application in China

In general, pesticides are extensively used by farmers in China. The means of the measured participant characteristics are similar to that of the average farmer in China (S4 Table). On average, participants in the present study applied pesticides on 12.8 separate occasions (total amount of 11.5 kg) in 2012 (Table 1). About 14% of the participants took protective measures (e.g., wearing masks and/or gloves) during pesticide application (Table 1).

**Table 1:** Average Pesticide Applications Per Person in 2012 and Percentage of Participants Reported Poisoning in 2009–2011.

Province	Time or frequencies	Total hours	Total amount of pesticide used (kg)	Percentage of wearing masks and/or gloves	Percentage of farmers reported poisoning in 2009–2011
**Average**	12.8	43.9	11.5	14	13
**Guangdong**	15.9	47.3	13.4	17	23
**Jiangxi**	14.7	56.3	8.9	11	8
**Hebei**	8.2	30.0	12.0	13	8

Data are from authors’ survey.

There were 183 varieties of pesticides applied by the farmers between the two rounds of health investigations. Most of them are pyrethroid, organophosphates and others. The high toxicity and heavy application of these pesticides produced effects on farmers’ health. The survey results show that 13% of participants had suffered from at least one acute health problem during pesticide application in the farm fields during 2009–2011 (Table 1). The percentage of participants who suffered from such health problems in Guangdong (23%) is higher than that of Jiangxi and Hebei (8%). This may be explained by the fact that farmers in Guangdong used a greater amount of pesticides than those in the other two provinces (Table 1).

### Long-term effects

Table 2 shows the results of two rounds of health investigations. CBC analysis in the first round showed that 70.25% of participants had at least one abnormal indicator (Table 2). Blood chemistry tests indicated abnormal renal function in 5.79% of subjects, and aberrant hepatic function, electrolyte levels, vitamin levels, and fasting plasma glucose levels in 10%–15% of subjects. Nerve conduction studies showed high percentages of abnormal DML (45.93%). Similar findings were observed in the TNSc (45.71%) and MMSE (20.82%).

**Table 2:** Percentage (%) of Abnormalities in Health Assessments in 2012.

Indicators	First round	Second round
**Blood examinations**		
***Complete blood count***	70.25	95.40
***Blood chemistry tests***		
Hepatic function	10.33	30.13
Renal function	5.79	20.92
Electrolytes	9.50	25.94
Vitamins	14.88	8.37
Fasting plasma glucose	13.22	5.44
C-reactive protein	6.61	2.93
**Nerve conduction studies**		
***Conduction velocity***	19.51	18.26
Motor nerves	10.57	10.37
Sensory nerves	12.60	12.86
Distal motor latency	45.93	31.54
***Amplitude***	10.57	9.13
Motor nerves	8.13	5.39
Sensory nerves	3.25	4.56
**Neurological examinations**		
TNSc	45.71	31.93
MMSE	20.82	10.50

Details of these results are presented in S5 Table. Data are from authors’ survey.

The number of abnormal health indicators increased significantly in the second round of investigation. For example, results of CBC analysis showed that nearly all participants (95.40%) examined had at least one abnormal indicator in the second round (Table 2). A large percentage of subjects also demonstrated abnormalities in hepatic function, renal function, electrolyte balance, and TNSc in the second round.

**Fig 1:**
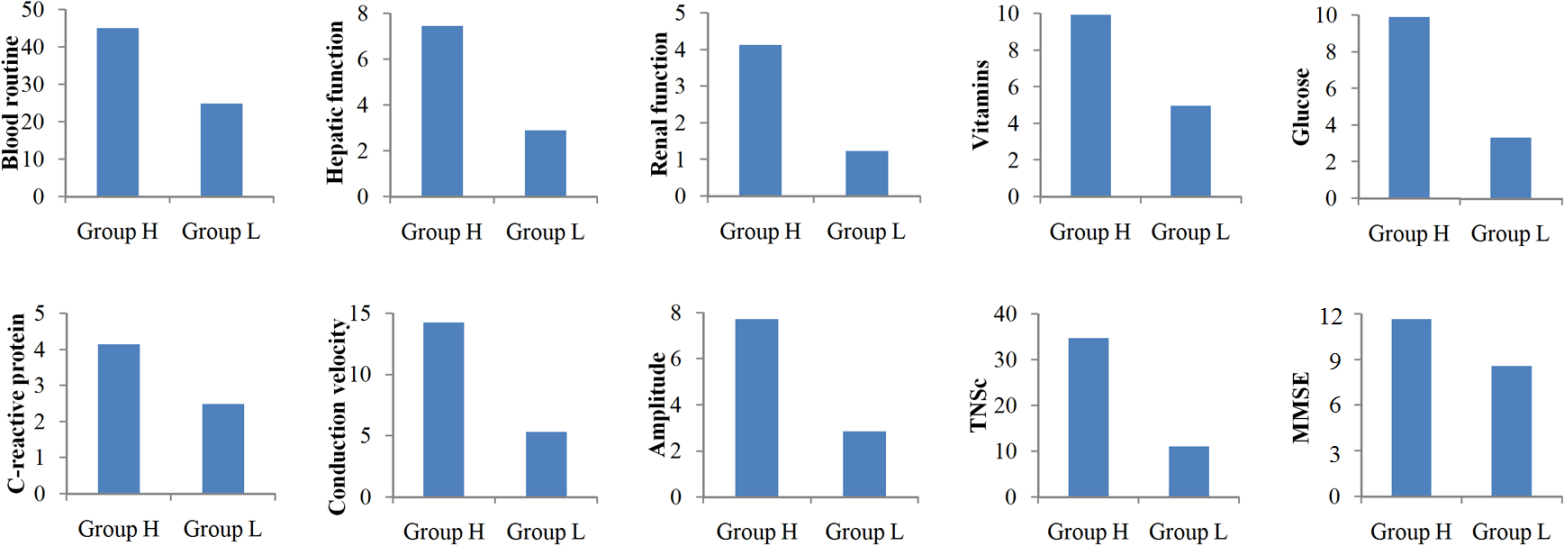
Percentage of abnormal cases by Group H and Group L in 2009–2011. The eight bar charts showed that the percentages of abnormal cases of major health indicators, including blood routine, hepatic function, renal function, vitamins, glucose, C-reactive protein, TNSc, MMSE, conduction velocity and amplitude, were higher in Group H than in Group L.

Fig 1 presents the percentages of abnormal cases of major health indicators for Group H and Group L in 2009–2011. The results show a consistent positive relationship between abnormal test results and intensity of pesticide use. This indicates that participants more frequently exposed to pesticides may suffer from CBC, hepatic, renal, glucose metabolic, peripheral, and central neurological ailments.

Multivariate regression analysis was conducted to control for the impact of other factors. Results showed significant differences in five health indicators between Group H and Group L (Table 3), while no difference was found in the other indicators. Specifically, participants with greater pesticide exposure had higher white blood cell counts, but lower creatinine and potassium levels. In addition, Group H demonstrated lower tibial nerve proximal and distal CMAP amplitude.

**Table 3:** Estimated Long-term Health Consequences of Pesticide Exposure among Farmers in China, 2009–2011 (OLS Estimation).

Variables	WBC		Cr		K		TNPCMAPA		TNDCMAPA	
**Pesticide application:**										
Group H	0.64 (0.03, 1.26)*		-5.32 (-8.91, -1.73)**		-0.28 (-0.48, -0.08)**		-1.35 (-2.61, -0.10)**		-1.53(-3.04, -0.02)*	
**Control variables:**										
Female	0.35 (-0.41, 1.12)		-18.00 (-22.43, -13.56)**		-0.02 (-0.27, 0.23)		-0.59 (-2.14, 0.95)		0.07 (-1.79, 1.93)	
Age (year)	-0.01 (-0.04, 0.01)		0.17 (0.04, 0.31*		0.00 (-0.01, 0.00)		-0.12 (-0.17, -0.07)**		-0.11 (-0.17, -0.05)**	
Height (cm)	-0.01 (-0.05, 0.04)		0.03 (-0.25, 0.31)		-0.01 (-0.02, 0.01)		0.03 (-0.07, 0.13)		0.12 (0.00, 0.24)*	
Weight (kg)	0.01 (-0.02, 0.04)		0.16 (0.01, 0.31*		0.01 (-0.00, 0.01)		-0.06 (-0.11, -0.01)*		-0.06 (-0.13, 0.00)	
Current smoker	0.59 (0.03, 1.16)*		-3.91 (-7.19, -0.63)*		0.16 (-0.02, 0.34)		-0.12 (-1.26, 1.03)		-0.37 (-1.75, 1.01)	
Current drinker	-0.07(-0.58, 0.44)		-0.65 (-3.60, 2.31)		0.20 (0.04, 0.37)*		-0.16 (-1.20, 0.88)		-0.61 (-1.87, 0.64)	
Constant	6.83 (-0.94, 14.60)		50.29 (5.19, 95.39)		5.47 (2.94, 7.99)**		17.40 (1.35, 33.44)*		4.19 (-15.12, 23.50)	
Observations	242		242		242		246		246	
R-squared	0.11		0.46		0.19		0.14		0.10	

Abbreviations: CI, confidence interval; Cr, creatinine; K, potassium; TNPCMAPA, tibial nerve proximal compound muscle action potential amplitude; TNDCMAPA, tibial nerve distal compound muscle action potential amplitude; WBC, white blood cell.

* and ** represent the levels of significance at 5% and 1%, respectively. Province dummy variables were included, but not reported.

However, while increases or decreases in the above health indicators were observed in Group H, some scores were still within the normal range. To address this issue, each of the health indicators was measured as a dummy (or binary) variable (abnormal = 1, normal = 0). A Probit model was used for estimation. The results showed a positive association between Group H and abnormalities in nerve conduction velocities (CVs), CMAP, and SNAP amplitudes; however, no relationship was found with other health indicators (S6 Table). A significant increase in sensory nerve abnormalities relative to motor nerve abnormalities was observed in peripheral nervous system (Table 4).

**Table 4:** Estimated Long-term Neurological Effects of Pesticide Exposure among Farmers in China, 2009–2011 (Dprobit Estimation).

Variables	Abnormal conduction Velocity			Abnormal amplitude		
	All nerves	Motor nerves	Sensory nerves	All nerves	Motor nerves	Sensory nerves
**Pesticide application:**						
Group H	0.16 (0.06, 0.25)**	0.05 (-0.01, 0.10)	0.10 (0.03, 0.18)**	0.05 (0.00, 0.10)*	0.02 (-0.03, 0.06)	0.03 (0.01, 0.06) *
**Control variables:**						
Female	-0.03 (-0.17, 0.12)	-0.02 (-0.10, 0.06)	0.00 (-0.11, 0.11)	0.00 (-0.08, 0.08)	0.03 (-0.06, 0.12)	-0.01 (-0.03, 0.01)
Age (year)	0.02 (0.01, 0.02)**	0.01 (0.00, 0.01)**	0.01 (0.01, 0.01)**	0.01 (0.00, 0.01)**	0.00 (0.00, 0.01)**	0.00 (-0.00, 0.00)*
Height (cm)	0.00 (-0.01, 0.01)	0.00 (-0.01, 0.01)	-0.00 (-0.01, 0.01)	0.00(-0.00, 0.01)	0.00 (-0.00, 0.01)	0.00 (-0.00, 0.00)
Weight (kg)	0.00 (-0.00, 0.01)	0.00 (-0.00, 0.00)	0.00 (-0.00, 0.00)	0.00 (-0.00, 0.00)	0.00 (-0.00, 0.00)	-0.00 (-0.00, 0.00)
Current smoker	-0.04 (-0.14, 0.07)	0.01 (-0.05, 0.07)	-0.05 (-0.13, 0.03)	0.02 (-0.03, 0.08)	0.05 (-0.00, 0.10)	-0.01 (-0.04, 0.01)
Current drinker	0.05 (-0.05, 0.16)	0.04 (-0.03, 0.11)	0.03 (-0.06, 0.11)	0.07 (0.00, 0.13)*	0.04 (-0.01, 0.10)	0.02 (-0.01, 0.05)
Observations	246	246	246	246	246	246

Abbreviations: CI, confidence interval

* and ** represent the levels of significance at 5% and 1%, respectively. Province dummy variables were included, but not reported.

### Short-term effects

The two rounds of health investigations in the present study allowed for the analysis of the short-term effects of pesticide exposure (frequency of pesticide application in the previous three days and in the previous 4–10 days) on health indicators. To accomplish this, a fixed effect model was used to control for individual differences, including the accumulated health effects of pesticide exposure prior to the first round of investigation. CBC analysis showed that pesticide application during the previous three days had significant effects on the majority of selected health indicators, such as monocytes, monocyte percentage, red blood cell, hemoglobin, hematocrit, mean corpuscular volume, mean corpuscular hemoglobin, mean corpuscular hemoglobin concentration, red cell distribution width coefficient of variation, platelet count, and platelet distribution width (Table 5). However, the effect of prior pesticide exposure on most indicators was absent (red blood cell, hemoglobin, platelet count, etc.) after 3 days.

**Table 5:** Estimated Short-term Health Effects of Pesticide Exposure on Blood Tests among Farmers in China, 2009–2011 (FE Estimation).

Dependent variables: ∆Indicator	Independent variables: ∆ frequencies of pesticide application			
	In past 3 days		In past 4–10 days	Constant
Mon	-0.11 (-0.15, -0.06)**	-0.05 (-0.09, 0.02)**		0.37 (0.34, 0.40)**
Monp	-1.98 (-2.73, -1.23)**	-0.94 (-1.50, -0.38)**		6.14 (5.66, 6.62)**
RBC	-0.17 (-0.25, -0.09) **	-0.03 (-0.09, 0.03)		4.69 (4.64, 4.75)**
Hb	-2.01 (-3.87, -0.16)*	-1.26 (-2.64, 0.12)		144.30 (143.1, 145.5)**
Hct	-0.88 (-1.63, -0.14)*	0.08 (-0.47, 0.64)		42.70 (42.22, 43.17)**
MCV	1.66 (0.72, 2.60)**	0.83 (-0.13, 1.52)*		91.62 (91.02, 92.22)**
MCH	0.70 (0.33, 1.06)**	-0.08 (-0.35, 0.19)		30.96 (30.72, 31.19)**
MCHC	2.27 (-0.25, 4.80)	-3.76 (-5.63, -1.88) **		337.17 (335.55, 338.78)**
RDW_CV	-0.16 (-0.33, 0.01)*	0.13 (0.01, 0.25)*		12.56 (12.45, 12.66)**
PLT	-8.67 (-16.53, -0.81)*	-3.47 (-9.30, 2.36)		215.62 (210.59, 220.65)**
PDW	0.75 (0.36, 1.14)**	0.21 (-0.07, 0.50)		14.65 (14.40, 14.90)**
ALT	4.54 (2.64, 6.43)**	-0.45 (-1.86, 0.95)		21.28 (20.06, 22.49)**
AST	3.59 (1.76, 5.43)**	-0.82 (-2.18, 0.53)		24.01 (22.84, 25.18)**
CHE	-355.24(-534.1, -176.4) **	-237.37(-370.1, -104.7)**		8641.03(8526.6, 8755.5)**
TP	-3.56 (-4.79, -2.32)**	-0.48 (-1.40, 0.43)		74.58 (73.78, 75.37)**
BUN	0.22 (0.02, 0.43)*	0.02 (-0.13, 0.18)		5.25 (5.12, 5.38)**
Na	-0.78 (-1.29, -0.27)**	-0.42 (-0.79, -0.04)*		141.83 (141.51, 142.16)**
P	0.07 (0.02, 0.12)**	-0.04 (-0.07, -0.005)*		1.24 (1.21, 1.27)**
VB_12_	-4.14 (-60.85, 52.56)	57.52 (15.46, 99.59) **		459.25 (422.98, 495.52) **
FPG	-0.25 (-0.40, -0.10)**	-0.17 (-0.28, -0.06)**		5.35 (5.25, 5.44)**

Abbreviations: ALT, alanine aminotransaminase; AST, aspartate aminotransferase; BUN, blood urea nitrogen; CHE, cholinesterase; CI, confidence interval; FPG, fasting plasma glucose; Hb, haemoglobin; Hct, hematocrit; Mon, monocytes; Monp, monocyte percentage; MCH, mean corpuscular haemoglobin; MCHC, mean corpuscular haemoglobin concentration; MCV, mean corpuscular volume; Na, Sodium; P, inorganic phosphorus; PDW, platelet distribution width; PLT, platelet count; RBC, red blood cell; RDW_CV, red cell distribution width coefficient of variation; TP, total protein; VB_12_, Vitamin B_12_.

* and ** represent the levels of significance at 5% and 1%, respectively.

Similar results were observed in the analysis of short-term health effects of pesticide exposure on blood chemistry tests (Table 5) and nerve conduction studies (Table 6). Blood chemistry results from three days post-exposure showed a significant increase in liver enzyme, blood urea nitrogen, and inorganic phosphorus levels; and a decrease in plasma cholinesterase, total protein, sodium, and fasting plasma glucose levels. However, these effects were not observable at 4–10 days post-exposure. Three CV indicators (median motor, ulnar motor, and ulnar sensory) were significantly increased at three days post-exposure, but not at 4–10 days post-exposure. The effects of pesticide exposure on health indicators related to DMLs (ulnar nerve) and amplitudes of CMAP (median, ulnar, peroneal) and SNAP (median, ulnar) were negative, which differs from the positive effects on CVs; however, the impact patterns over time are similar.

**Table 6:** Estimated Short-term Health Effects of Pesticide Exposure on Nerve Conduction among Farmers in China, 2009–2011 (FE Estimation).

Dependent variables: ∆Indicator	Independent variables: ∆ frequencies of pesticide application		
	In past 3 days	In past 4–10 days	Constant
Median MCV	0.74 (0.10, 1.38)*	-0.15 (-0.66, 0.37)	59.01 (58.55, 59.46)**
Ulnar MCV	0.62 (0.03, 1.21)*	0.28 (-0.20, 0.75)	57.18 (56.76, 57.60)**
Ulnar SCV	0.89 (0.14, 1.64)*	0.32 (-0.29, 0.92)	53.46 (52.92, 53.99)**
Median DML	-0.06 (-0.15, 0.03)	-0.07 (-0.15, -0.001)*	3.46 (3.40, 3.53)**
Ulnar DML	-0.16 (-0.25, -0.07)**	-0.04 (-0.12, 0.04)	2.83 (2.76, 2.89)**
Median proximal CMAP	-0.70 (-1.17, -0.23)**	-0.12 (-0.50, 0.26)	13.27 (12.94, 13.61)**
Median distal CMAP	-0.61 (-1.07, -0.14)*	0.02 (-0.35, 0.39)	13.76 (13.43, 14.09)**
Ulnar proximal CMAP	-0.74 (-1.06, -0.42)**	-0.20 (-0.46, 0.06)	12.25 (12.03, 12.48)**
Ulnar distal CMAP	-0.45 (-0.74, -0.16)**	-0.20 (-0.44, 0.04)	12.89 (12.68, 13.10)**
Peroneal proximal CMAP	-0.12 (-0.39, 0.14)	-0.23 (-0.44, -0.01)*	6.79 (6.60, 6.98)**
Median SNAP	-0.75 (-1.06, -0.45)**	0.15 (-0.09, 0.40)	8.20 (7.98, 8.41)**
Ulnar SNAP	-0.33 (-0.57, -0.09)**	-0.07 (-0.26, 0.12)	6.19 (6.02, 6.35)**

Abbreviations: CI, confidence interval; CMAP, compound muscle action potential; DML, distal motor latency; MCV, motor conduction velocity; SCV, sensory conduction velocity; SNAP, sensory nerve action potential.

* and ** represent the levels of significance at 5% and 1%, respectively.

## Discussion

### Nervous system

This study was the first to comprehensively examine the human health effects of frequent pesticide exposure in China. Organophosphate-induced delayed polyneuropathy (OPIDN) might develop after acute organophosphates exposure [12], but there was no evidence of such dysfunction after prolonged low-level exposure to organophosphates or other pesticide species [23]. To detect clinical evidence of polyneuropathy, we used the total neuropathy score, which was widely used in studies of toxic neuropathies [22] and other distal length-dependent neuropathies [24]. Its reduced version (the TNSc) included only items of symptoms and signs revealed by clinical neurological examinations. This score graded and combined the severity of motor, sensory, and autonomic symptoms and neurological signs, and prevented the diluting effect of other non-distal symptoms such as radiculopathy and entrapment syndromes. We found that sensory abnormalities were much more common than motor abnormalities (Table 4). However, the score was not sensitive enough to detect neurological function changes among different exposure levels and over different periods.

Nerve conduction studies were used as objective measurements in the present research. We tested nerves on the non-dominant side to minimize the potential confound of nerve injury due to work activities rather than pesticides. In our estimation of long-term effects, the proximal and distal CMAP of the tibial nerve, which reflects axonal function in the lower limbs, decreased in participants with a higher exposure level, but most of them did not meet the abnormal criteria. Abnormal electrophysiological profiles (CV, CMAP, and SNAP amplitude) were seen in the higher exposure group, and mostly in sensory nerves. These findings suggest that prolonged, low-level pesticide exposure can also cause peripheral neurotoxicity, especially in sensory nerves, which was consistent with the TNSc findings. This form of sensory neuropathy is different from OPIDN, in which prominent sensory loss is not a main feature [25]. Although OP compound exposure has been associated with sensory neuropathy based on neurological symptoms, neurological examinations or quantitative sensory testing [26], some studies did not find evidence of sensory neuropathy among workers with long-term occupational exposure to Chlorpyrifos, an OP [27]. Our results are positive and robust for two reasons. First, the present study examined a larger sample of participants that were exposed to higher pesticide levels; secondly, we measured neurological variables at consistent times following exposure. Since nerve conduction measurements merely reflect abnormal large fiber populations, we need to further evaluate small nerve fibers, which mediate pain, thermal sensation, and autonomic function.

The present study also contributes to better understanding of the short-term health effects of pesticide exposure in crop production. We found that most effects were evident within 3 days post-exposure, but significantly attenuated after 4 days. It is presumed that these short-term reversible alterations in both amplitude and CV might be due to transient actions of pesticides on ion channels in peripheral nerves, ultimately resulting in altered intracellular ionic levels. Such alterations may trigger secondary changes that induce cytotoxicity in axons and neuronal cell bodies, similar to chemotherapy-induced peripheral neurotoxicity [28]. However, the precise mechanisms require further study.

We used the MMSE, the most common cognitive screening assessment tool, to assess changes in the central nervous system. As shown in Fig 1, frequent pesticide exposure tended to increase the likelihood of an abnormal MMSE score; however, this association was not significant. The lack of significance may have been due to the smaller sample size of our study compared with other studies, particularly one that indicated an increased risk of Alzheimer disease among pesticide-exposed individuals [29]. Nevertheless, this preliminary finding necessitates further investigation into this problem.

### Hematological system

In the hematological system, our study is consistent with some recent findings—alterations in CBC parameters are related to pesticides exposure [30]. Yet, it provides better understanding of pesticide health effects in long- and short-term alterations. Specifically, only white blood cell count remains significantly increased over time. A meta-analysis showed a positive relationship between pesticide exposure and hematopoietic cancers, including non-Hodgkin’s lymphoma [31]. Although the increase in the number of white blood cells in peripheral blood was still within the normal range, it might suggest that pesticides affect the hematopoietic function of bone marrow. On the contrary, short-term pesticide exposure decreased monocytes, hemoglobin, and platelets, which suggests a direct toxic effect of pesticides on peripheral blood cells.

With regard to the metabolic panel, our results showed that pesticide exposure led to acute hepatic dysfunction and FPG reduction, which was opposite to previous reports of pesticide-associated hyperglycemia [15]. However, there were some reports of hypoglycemia following pesticide exposure [32]. This paradoxical phenomenon might suggest that different types of pesticides exert different effects. Electrolytes, including potassium, sodium, and inorganic phosphorus, were also affected either in long-term or in short-term. To date, only one study has shown electrolyte alterations following pesticide (acetamiprid) exposure [33], and the mechanism and significance of this has not yet been explored. Finally, renal function was also both acutely and chronically affected, and these abnormalities have been shown in previous studies [34].

### Strengths and limitations

We selected three provinces that representing high (Guangdong), middle (Jiangxi), and low (Hebei) levels of pesticide application provinces in China in order to have large variations on pesticide used based on farmers’ average pesticide use by province from the surveys conducted by the National Development and Reform Commission, but after we conducted our surveys in the selected counties in the above three provinces, we found that farmers used less pesticide in Jiangxi than that in Hebei. However, this does not affect our analysis because our data show that there were large variations on pesticide uses among our samples among provinces. In contrast to earlier studies, the present study thoroughly evaluated various health indicators, used novel methodology for analyzing the duration and extent of pesticide exposure, contained a large sample size, and had a high response rate. Based on our survey, the major crops are: rice, vegetables, and orchards in Guangdong; rice, cotton, and rapeseed in Jiangxi; and wheat, maize, and cotton in Hebei. Moreover, the control was unique. Farmers with lower level pesticides exposure were used as control for long-term health effects estimation, while self-control was used when short-term effects were evaluated. Yet, several limitations are noticed. First, the base for pesticide application was established based on farmers’ recall of their pesticide application in 2009–2011. Such measure is subject to recall errors and may not represent participants’ lifetime exposure of pesticide. For example, participants who sprayed pesticides frequently before 2009 might have sprayed less than 50 times in 2009 to 2011, and vice versa. Secondly, to estimate the chronic health effects of pesticide exposure, such as cancer, Parkinson’s disease, and Alzheimer’s disease, it is necessary to follow subjects for an even longer time period. Finally, the pesticide-associated changes in the health indicators measured in the present study demonstrate the general effects of multiple pesticides, but other indicators should additionally be examined to yield insight into specific disorders. Similarly, the effects of each kind of pesticide, particularly high toxic pesticides, should be further analyzed.

## Conclusions

In summary, we found that there were extensive long-term and short-term health effects in farmers exposed to pesticides. The former involved peripheral nervous system, white blood cells, liver, electrolytes, and the latter involved blood cells, liver, kidney, electrolytes and peripheral nervous system. Future studies are necessary to elucidate the mechanisms of these effects. It should be indicated that the high toxic pesticides, such as omethoate and other organophosphates are still used extensively in the crops production. Pyrethroid and some types of median toxic pesticides are more widely used. The high and median toxic pesticides are most harmful to the farmers. The government should revise the policy to reduce the pesticides production and application.

## Supporting Information

S1 Table Definition and normal range of all the health indicators. (DOCX)

S2 Table Clinical total neuropathy score (TNSc). (DOCX)

S3 Table Number of farmers participated in health investigations. (DOCX)

S4 Table Basic characteristics of sample farmers in 2011. (DOCX)

S5 Table Mean and abnormal cases in two rounds of health investigations. (DOCX)

S6 Table Estimated results of the long-term health effects (Dprobit estimation). (DOCX)

S7 Table Estimated results of the short-term effects adjusting for season (FE estimation). (DOCX)

S8 Table Estimated results of the short-term effects on blood routine adjusting for regions (FE estimation). (DOCX)

S9 Table Estimated results of the short-term effects on blood biochemistry and C-reactive protein adjusting for regions (FE estimation). (DOCX)

S10 Table Estimated results of the short-term effects on farmer’s nerve conduction studies and neurological examination adjusting for regions (FE estimation). (DOCX)
