# Supplementary material for: Long- and Short-Term Health Effects of Pesticide Exposure: A Cohort Study from China
Source: PLoS One. 2015 Jun 4;10(6):e0128766. doi: 10.1371/journal.pone.0128766 (PMC4456378; doi:10.1371/journal.pone.0128766)
Supplement: S1 Table — (DOCX) [file pone.0128766.s001.docx]

**S1 Table. Definition and normal range of all the health indicators**

| **Examined indicator** | **Definition** | **Unit** | **Normal range** |
| --- | --- | --- | --- |
| **Blood routine** |  |  |  |
| WBC | White blood cell | 10^9^/L | 4.0-10.0 |
| Neu | Neutrophil | 10^9^/L | 2.0-7.0 |
| Lym | Lymphocyte | 10^9^/L | 0.8-4.0 |
| Mon | Monocyte | 10^9^/L | 0.12-1.00 |
| Neup | Neutrophil percentage | % | 55.0-75.0 |
| Lymp | Lymphocyte percentage | % | 20-40 |
| Monp | Monocyte percentage | % | 3.0-10.0 |
| RBC | Red blood cell | 10^12^/L | 3.5-5.0(female)  4.0-5.5(male) |
| Hb | Hemoglobin | g/L | 110-150(female)  120-160(male) |
| Hct | Hematocrit | % | 37-43(female)  42-49(male) |
| MCV | Mean corpuscular volume | fl | 80-98 |
| MCH | Mean corpuscular hemoglobin | pg | 27.0-35.0 |
| MCHC | Mean corpuscular hemoglobin concentration | g/L | 320-362 |
| RDW_CV | Red cell distribution width coefficient of variation | % | 11.0-16.0 |
| PLT | Platelet count | 10^9^/L | 100-300 |
| MPV | Mean volume of platelets | fl | 7.6-13.6 |
| PDW | Platelet distribution width | fl | 9.0-17.0 |
| **Blood biochemistry** |  |  |  |
| ***Hepatic function*** |  |  |  |
| ALT | Alanine aminotransferase | U/L | 0-40 |
| AST | Aspartate aminotransferase | U/L | 0-40 |
| CHE | Cholinesterase | U/L | 5320-12920 |
| TP | Total protein | g/L | 66.0-87.0 |
| ***Renal function*** |  |  |  |
| Urea | Urea | mmol/L | 1.70-8.30 |
| Cr | Creatinine | *μ*mol/L | 44-80(female)  62-106(male) |
| ***Electrolytes*** |  |  |  |
| Na | Sodium | mmol/L | 136-145 |
| K | Potassium | mmol/L | 3.50-5.20 |
| P | Inorganic phosphorus | mmol/L | 0.87-1.45 |
| ***Vitamins*** |  |  |  |
| VB_12_ | Vitamin B_12_ | ng/L | 191-946 |
| Folic acid | Folic acid | *μ*g/L | 3.10-17.50 |
| ***Glucose*** |  |  |  |
| Glu | Glucose | mmol/L | 3.90-6.10 |
| ***C-reactive protein*** |  |  |  |
| CRP | C-reactive protein | mg/L | 0.00-5.00 |
| **Conduction velocity** |  |  |  |
| ***Motor nerves*** |  |  |  |
| MNMCV | Median Nerve Motor Conduction Velocity | m/s | ≥50 |
| UNMCV | Ulnar Nerve Motor Conduction Velocity | m/s | ≥50 |
| TNMCV | Tibial Nerve Motor Conduction Velocity | m/s | ≥40 |
| PNMCV | Common Peroneal Nerve Motor Conduction Velocity | m/s | ≥45 |
| ***Sensory nerves*** |  |  |  |
| MNSCV | Median Nerve Sensory Conduction Velocity | m/s | ≥50 |
| UNSCV | Ulnar Nerve Sensory Conduction Velocity | m/s | ≥50 |
| SNSCV | Sural Nerve Sensory Conduction Velocity | m/s | ≥50 |
| ***Distal motor latency*** |  |  |  |
| MNDML | Median Nerve Distal Motor Latency | ms | ≤3.63 |
| UNDML | Ulnar Nerve Distal Motor Latency | ms | ≤3.07 |
| TNDML | Tibial Nerve Distal Motor Latency | ms | ≤4.80 |
| PNDML | Common Peroneal Nerve Distal Motor Latency | ms | ≤4.50 |
| **Amplitude** |  |  |  |
| ***Motor nerves*** |  |  |  |
| MNPCMAPA | Median Nerve Proximal Compound Muscle Action Potential Amplitude | mV | ≥5.0 |
| MNDCMAPA | Median Nerve Distal Compound Muscle Action Potential Amplitude | mV | ≥5.0 |
| UNPCMAPA | Ulnar Nerve Proximal Compound Muscle Action Potential Amplitude | mV | ≥5.0 |
| UNDCMAPA | Ulnar Nerve Distal Compound Muscle Action Potential Amplitude | mV | ≥5.0 |
| TNPCMAPA | Tibial Nerve Proximal Compound Muscle Action Potential Amplitude | mV | ≥4.8 |
| TNDCMAPA | Tibial Nerve Distal Compound Muscle Action Potential Amplitude | mV | ≥4.8 |
| PNPCMAPA | Common Peroneal Nerve Proximal Compound Muscle Action Potential Amplitude | mV | ≥2.0 |
| PNDCMAPA | Common Peroneal Nerve Distal Compound Muscle Action Potential Amplitude | mV | ≥2.0 |
| ***Sensory nerves*** |  |  |  |
| MNSNAPA | Median Nerve Sensory Nerve Action Potential Amplitude | mV | ≥2.0 |
| UNSNAPA | Ulnar Nerve Sensory Nerve Action Potential Amplitude | mV | ≥2.0 |
| SNSNAPA | Sural Nerve Sensory Nerve Action Potential Amplitude | mV | ≥2.0 |
| **Neurological examinations** |  |  |  |
| ***TNSc* ^1^** |  |  | ≥2 |
| Sensory symptoms | Grading of paraesthesia |  | 0-4 |
| Motor symptoms | Grading of limb weakness |  | 0-4 |
| Autonomic symptoms | Grading of autonomic symptoms, including sweating abnormalities, dizziness and fainting, urinary problems, difficulty digesting food, and impotence |  | 0-4 |
| Pin sensibility | Grading of decreased pin sensibility |  | 0-4 |
| Vibration sensibility | Grading of decreased vibration sensibility |  | 0-4 |
| Strength | Grading of muscle strength according to the muscle with the worst result |  | 0-4 |
| DTR | Grading of decreased deep tendon reflex |  | 0-4 |
| ***MMSE*** |  |  |  |
| MMSE | Mini-Mental State Examination |  | >17(education=0),>20(0<education≤6),>26(education>6) |
| **General examinations** |  |  |  |
| Height | Height | cm |  |
| Weight | Weight | kg |  |
| Blood pressure | Blood pressure | mmHg |  |
| Urinalysis | Urinalysis |  |  |
| ECG | Electrocardiogram |  |  |
| Ultrasound | Ultrasound |  |  |

^1^ See Table S2: Clinical total neuropathy score (TNSc).
