# Supplementary material for: Long- and Short-Term Health Effects of Pesticide Exposure: A Cohort Study from China
Source: PLoS One. 2015 Jun 4;10(6):e0128766. doi: 10.1371/journal.pone.0128766 (PMC4456378; doi:10.1371/journal.pone.0128766)
Supplement: S2 Table — (DOCX) [file pone.0128766.s002.docx]

**S2 Table.** **Clinical total neuropathy score (TNSc)**

| **Parameters** | **Score** | | | | |
| --- | --- | --- | --- | --- | --- |
|  | **0** | **1** | **2** | **3** | **4** |
| Sensory symptoms | None | Symptoms limited finger or toes | Symptoms extend to ankle or wrist | Symptoms extend to knee or elbow | Symptoms above knees or elbow or functionally disabling |
| Motor symptoms | None | Slightly difficulty | Moderate difficulty | Require held/assistance | Paralysis |
| Autonomic symptoms ^1^ | 0 | 1 | 2 | 3 | 4 or 5 |
| Pin sensibility | Normal | Reduced in fingers/toes | Reduced up to wrist/ankle | Reduced up to elbow/knee | Reduced to above elbow/knee |
| Vibration sensibility | Normal | Reduced in fingers/toes | Reduced up to wrist/ankle | Reduced up to elbow/knee | Reduced to above elbow/knee |
| Strength ^2^ | Normal | Mild weakness  (MRC 4) ^3^ | Moderate weakness  (MRC 3) | Severe weakness  (MRC 2) | Paralysis  (MRC 0-1) |
| DTR | Normal | Ankle reflex reduced | Ankle reflex absent | Ankle reflex absent, others reduced | All reflexes absent |

^1^ Number of symptoms reported by farmers.

^2^ In the muscle with the worst result.

^3^ MRC: Medical Research Council (muscles/groups evaluated: toe extensors, toe flexors, triceps surae, tibialis anterior, quadriceps femoris, iliopsoas in the lower limbs and finger flexors, finger extensors, wrist flexors, wrist extensors, biceps brachii and triceps brachii in the upper limbs).
