# Supplementary material for: Long- and Short-Term Health Effects of Pesticide Exposure: A Cohort Study from China
Source: PLoS One. 2015 Jun 4;10(6):e0128766. doi: 10.1371/journal.pone.0128766 (PMC4456378; doi:10.1371/journal.pone.0128766)
Supplement: S3 Table — (DOCX) [file pone.0128766.s003.docx]

**S3 Table. Number of farmers participated in health investigations**

|  | **First round** | **Second round** | **Both rounds** |
| --- | --- | --- | --- |
| Blood examinations | 242 | 239 | 215 |
| Nerve conduction studies | 246 | 241 | 221 |
| Neurological examinations | 245 | 238 | 220 |

Data are from authors’ survey.
