# Supplementary material for: Long- and Short-Term Health Effects of Pesticide Exposure: A Cohort Study from China
Source: PLoS One. 2015 Jun 4;10(6):e0128766. doi: 10.1371/journal.pone.0128766 (PMC4456378; doi:10.1371/journal.pone.0128766)
Supplement: S4 Table — (DOCX) [file pone.0128766.s004.docx]

**S4 Table.** **Basic characteristics of sample farmers in 2011**

|  | **Mean** | **St. Error** | **Min** | **Max** |
| --- | --- | --- | --- | --- |
| Age (year) | 51.46 | 10.13 | 24 | 76 |
| Female (yes=1, no=0) | 0.28 | 0.45 | 0 | 1 |
| Education (year) | 7.19 | 3.70 | 0 | 15 |
| Height (cm) | 164.80 | 7.25 | 140 | 182 |
| Weight (kg) | 64.00 | 11.74 | 45 | 100 |
| Smoking (yes=1, no=0) | 0.47 | 0.50 | 0 | 1 |
| Drinking (yes=1, no=0) | 0.42 | 0.49 | 0 | 1 |
| Family population (number) | 4.23 | 1.61 | 1 | 9 |
| Cultivate land (mu) | 10.75 | 13.97 | 0 | 170 |

Data are from authors’ survey. The total number of sample is 246.
