# Supplementary material for: Long- and Short-Term Health Effects of Pesticide Exposure: A Cohort Study from China
Source: PLoS One. 2015 Jun 4;10(6):e0128766. doi: 10.1371/journal.pone.0128766 (PMC4456378; doi:10.1371/journal.pone.0128766)
Supplement: S5 Table — (DOCX) [file pone.0128766.s005.docx]

**S5 Table.** **Mean and abnormal cases in two rounds of health investigations**

| **Indicators** | **In the first round of investigation** | | |  | **In the second round of investigation** | | |
| --- | --- | --- | --- | --- | --- | --- | --- |
|  | **Mean** | **Abnormal cases** | **Percentage of abnormal cases (%)** |  | **Mean** | **Abnormal cases** | **Percentage of abnormal cases (%)** |
| **Blood routine** | **-** | **170** | **70.25** |  | **-** | **228** | **95.4** |
| WBC | 6.19 | 12 | 4.96 |  | 6.29 | 25 | 10.46 |
| Neu | 3.65 | 3 | 1.24 |  | 3.72 | 18 | 7.53 |
| Lym | 2.10 | 1 | 0.41 |  | 2.03 | 4 | 1.67 |
| Mon | 0.45 | 3 | 1.24 |  | 0.16 | 111 | 46.44 |
| Neup | 59.24 | 68 | 28.10 |  | 58.77 | 87 | 36.40 |
| Lymp | 33.42 | 41 | 16.94 |  | 32.62 | 51 | 21.34 |
| Monp | 7.34 | 17 | 7.02 |  | 2.46 | 154 | 64.44 |
| RBC | 4.76 | 31 | 12.81 |  | 4.45 | 30 | 12.55 |
| Hb | 144.82 | 43 | 17.77 |  | 140.87 | 33 | 13.81 |
| Hct | 42.92 | 78 | 32.23 |  | 41.94 | 101 | 42.26 |
| MCV | 90.94 | 21 | 8.68 |  | 94.60 | 79 | 33.05 |
| MCH | 30.62 | 16 | 6.61 |  | 31.76 | 24 | 10.04 |
| MCHC | 335.88 | 16 | 6.61 |  | 335.84 | 35 | 14.64 |
| RDW_CV | 12.68 | 3 | 1.24 |  | 12.43 | 19 | 7.95 |
| PLT | 208.98 | 22 | 9.09 |  | 212.97 | 41 | 17.15 |
| MPV | 11.04 | 0 | 0.00 |  | 10.19 | 13 | 5.44 |
| PDW | 14.08 | 9 | 3.72 |  | 15.99 | 5 | 2.09 |
| **Blood biochemistry** |  |  |  |  |  |  |  |
| ***Hepatic function*** | **-** | **25** | **10.33** |  | **-** | **72** | **30.13** |
| ALT | 20.07 | 13 | 5.37 |  | 24.54 | 24 | 10.04 |
| AST | 22.82 | 5 | 2.07 |  | 26.29 | 18 | 7.53 |
| CHE | 8656.47 | 5 | 2.07 |  | 8085.88 | 6 | 2.51 |
| TP | 76.66 | 7 | 2.89 |  | 69.62 | 39 | 16.32 |
| ***Renal function*** | **-** | **14** | **5.79** |  | **-** | **50** | **20.92** |
| Urea | 5.13 | 2 | 0.83 |  | 5.52 | 15 | 6.28 |
| Cr | 72.71 | 12 | 4.96 |  | 75.58 | 39 | 16.32 |
| ***Electrolytes*** | **-** | **23** | **9.50** |  | **-** | **62** | **25.94** |
| Na | 141.97 | 4 | 1.65 |  | 140.70 | 0 | 0.00 |
| K | 4.66 | 20 | 8.26 |  | 4.43 | 0 | 0.00 |
| P | 1.18 | 2 | 0.83 |  | 1.30 | 62 | 25.94 |
| ***Vitamins*** | **-** | **36** | **14.88** |  | **-** | **20** | **8.37** |
| VB_12_ | 486.32 | 21 | 8.68 |  | 484.57 | 18 | 7.53 |
| Folic acid | 8.23 | 16 | 6.61 |  | 10.52 | 4 | 1.67 |
| ***Glucose*** | **5.44** | **32** | **13.22** |  | **4.90** | **13** | **5.44** |
| Glu | 5.44 | 32 | 13.22 |  | 4.90 | 13 | 5.44 |
| ***C-reactive protein*** | **1.58** | **16** | **6.61** |  | **1.19** | **7** | **2.93** |
| CRP | 1.58 | 16 | 6.61 |  | 1.19 | 7 | 2.93 |
| **Conduction velocity** | **-** | **48** | **19.51** |  | **-** | **44** | **18.26** |
| ***Motor nerves*** | **-** | **26** | **10.57** |  | **-** | **25** | **10.37** |
| MNMCV | 58.45 | 2 | 0.81 |  | 59.71 | 1 | 0.41 |
| UNMCV | 57.19 | 4 | 1.63 |  | 57.89 | 4 | 1.66 |
| TNMCV | 47.70 | 2 | 0.81 |  | 47.74 | 3 | 1.24 |
| PNMCV | 48.53 | 21 | 8.54 |  | 48.59 | 23 | 9.54 |
| ***Sensory nerves*** | **-** | **31** | **12.60** |  | **-** | **31** | **12.86** |
| MNSCV | 58.43 | 13 | 5.28 |  | 58.26 | 15 | 6.22 |
| UNSCV | 53.81 | 18 | 7.32 |  | 54.15 | 18 | 7.47 |
| SNSCV | 60.45 | 0 | 0.00 |  | 59.65 | 0 | 0.00 |
| ***Distal motor latency*** | **-** | **113** | **45.93** |  | **-** | **76** | **31.54** |
| MNDML | 3.45 | 73 | 29.67 |  | 3.34 | 53 | 21.99 |
| UNDML | 2.87 | 61 | 24.80 |  | 2.64 | 16 | 6.64 |
| TNDML | 3.70 | 4 | 1.63 |  | 3.67 | 0 | 0.00 |
| PNDML | 3.72 | 18 | 7.32 |  | 3.73 | 20 | 8.30 |
| **Amplitude** | **-** | **26** | **10.57** |  | **-** | **22** | **9.13** |
| ***Motor nerves*** | **-** | **20** | **8.13** |  | **-** | **13** | **5.39** |
| MNPCMAPA | 13.63 | 1 | 0.41 |  | 12.23 | 0 | 0.00 |
| MNDCMAPA | 14.07 | 1 | 0.41 |  | 12.99 | 0 | 0.00 |
| UNPCMAPA | 12.42 | 2 | 0.81 |  | 11.37 | 0 | 0.00 |
| UNDCMAPA | 13.02 | 0 | 0.00 |  | 12.18 | 0 | 0.00 |
| TNPCMAPA | 11.31 | 7 | 2.85 |  | 11.18 | 6 | 2.49 |
| TNDCMAPA | 13.72 | 2 | 0.81 |  | 14.32 | 1 | 0.41 |
| PNPCMAPA | 6.82 | 12 | 4.88 |  | 6.48 | 9 | 3.73 |
| PNDCMAPA | 7.41 | 9 | 3.66 |  | 7.15 | 7 | 2.90 |
| ***Sensory nerves*** | **-** | **8** | **3.25** |  | **-** | **11** | **4.56** |
| MNSMAPA | 8.41 | 1 | 0.41 |  | 7.62 | 4 | 1.66 |
| UNSNAPA | 6.21 | 7 | 2.85 |  | 5.76 | 8 | 3.32 |
| SNSNAPA | 16.50 | 0 | 0.00 |  | 15.32 | 0 | 0.00 |
| **Neurological examinations examinations eexaminations** |  |  |  |  |  |  |  |
| ***TNSc*** | **2.07** | **112** | **45.71** |  | **1.32** | **76** | **31.93** |
| Sensory symptoms | 0.33 | 66 | 26.94 |  | 0.09 | 12 | 5.04 |
| Motor symptoms | 0.11 | 26 | 10.61 |  | 0.06 | 15 | 6.30 |
| Autonomic symptoms | 0.71 | 111 | 45.31 |  | 0.49 | 78 | 32.77 |
| Pin sensibility | 0.23 | 31 | 12.65 |  | 0.26 | 42 | 17.65 |
| Vibration sensibility | 0.36 | 45 | 18.37 |  | 0.08 | 14 | 5.88 |
| Strength | 0.04 | 9 | 3.67 |  | 0.05 | 11 | 4.62 |
| DTR | 0.29 | 36 | 14.69 |  | 0.28 | 34 | 14.29 |
| ***MMSE*** | **25.71** | **51** | **20.82** |  | **26.78** | **25** | **10.50** |
| MMSE | 25.71 | 51 | 20.82 |  | 26.78 | 25 | 10.50 |
| **General examinations** |  |  |  |  |  |  |  |
| Blood pressure | - | 111 | 40.96 |  | - | 87 | 31.41 |
| Urinalysis | - | 35 | 12.92 |  | - | 38 | 13.72 |
| ECG | - | 83 | 30.63 |  | - | 59 | 21.30 |
| Ultrasound | - | 85 | 31.37 |  | - | 93 | 33.57 |

Data are from authors’ survey.
