# Supplementary material for: Long- and Short-Term Health Effects of Pesticide Exposure: A Cohort Study from China
Source: PLoS One. 2015 Jun 4;10(6):e0128766. doi: 10.1371/journal.pone.0128766 (PMC4456378; doi:10.1371/journal.pone.0128766)
Supplement: S6 Table — (DOCX) [file pone.0128766.s006.docx]

**S6 Table. Estimated results of the long-term health effects (Dprobit estimation).**

|  | **Blood routine** | **Hepatic function** | **Renal function** | **Electrolytes** | **Vitamins** | **Glucose** | **C-reactive protein** | **TNSc** | **MMSE** | **Conduction velocity** | **Distal motor latency** | **Amplitude** |
| --- | --- | --- | --- | --- | --- | --- | --- | --- | --- | --- | --- | --- |
| **Pesticide application** |  |  |  |  |  |  |  |  |  |  |  |  |
| Group H (yes=1, no=0) | 0.01 | 0.02 | 0.05 | -0.06 | 0.01 | 0.04 | 0.04 | 0.09 | -0.07 | 0.16** | -0.05 | 0.05* |
|  | (0.08) | (0.05) | (0.03) | (0.05) | (0.06) | (0.05) | (0.03) | (0.09) | (0.06) | (0.05) | (0.09) | (0.03) |
| **Control variables** |  |  |  |  |  |  |  |  |  |  |  |  |
| Female(yes=1, no=0) | 0.13 | -0.01 | 0.04 | 0.02 | 0.01 | 0.03 | -0.04 | 0.18 | 0.08 | -0.03 | -0.08 | 0.00 |
|  | (0.09) | (0.06) | (0.05) | (0.06) | (0.07) | (0.07) | (0.04) | (0.11) | (0.07) | (0.07) | (0.10) | (0.04) |
| Age (year) | 0.01 | 0.00 | 0.00 | 0.00 | 0.00 | 0.01** | 0.00 | 0.00 | 0.005** | 0.02** | 0.01** | 0.01** |
|  | (0.00) | (0.00) | (0.00) | (0.00) | (0.00) | (0.00) | (0.00) | (0.00) | (0.00) | (0.00) | (0.00) | (0.00) |
| Height (cm) | -0.02* | -0.00 | 0.00 | -0.00 | -0.00 | -0.01 | -0.01 | -0.01 | -0.00 | 0.00 | 0.00 | 0.00 |
|  | (0.01) | (0.00) | (0.00) | (0.00) | (0.00) | (0.00) | (0.00) | (0.01) | (0.00) | (0.00) | (0.01) | (0.00) |
| Weight (kg) | 0.00 | 0.00 | -0.00 | 0.00 | -0.00 | 0.01** | 0.00 | -0.00 | -0.005* | 0.00 | 0.01 | 0.00 |
|  | (0.00) | (0.00) | (0.00) | (0.00) | (0.00) | (0.00) | (0.00) | (0.00) | (0.00) | (0.00) | (0.00) | (0.00) |
| Current smoker (yes=1, no=0) | 0.04 | 0.04 | -0.01 | 0.02 | -0.07 | -0.03 | -0.01 | 0.13 | -0.00 | -0.04 | 0.01 | 0.02 |
|  | (0.07) | (0.05) | (0.03) | (0.04) | (0.06) | (0.05) | (0.03) | (0.08) | (0.04) | (0.05) | (0.08) | (0.03) |
| Current drinker (yes=1, no=0) | 0.16** | -0.02 | -0.02 | 0.02 | -0.06 | 0.05 | 0.01 | -0.07 | -0.03 | 0.05 | 0.00 | 0.07* |
|  | (0.06) | (0.04) | (0.03) | (0.04) | (0.05) | (0.05) | (0.03) | (0.07) | (0.04) | (0.05) | (0.07) | (0.03) |
| **Observations** | 242 | 242 | 242 | 242 | 242 | 242 | 242 | 245 | 245 | 246 | 246 | 246 |

** and * indicate the statistically significant at 1% and 5%, respectively. Current smokers were those who had smoked ≥100 cigarettes or ≥20 pipes of tobacco over their lifetime and smoked at the time of the interview. Current drinkers were those who had at least one drink of any kind of alcoholic beverage in the past year.
