# Supplementary material for: Long- and Short-Term Health Effects of Pesticide Exposure: A Cohort Study from China
Source: PLoS One. 2015 Jun 4;10(6):e0128766. doi: 10.1371/journal.pone.0128766 (PMC4456378; doi:10.1371/journal.pone.0128766)
Supplement: S7 Table — (DOCX) [file pone.0128766.s007.docx]

**S7 Table. Estimated results of the short-term effects adjusting for season (FE estimation).**

| **Dependent variables:**  **∆Indicator** | **Independent variables: ∆ frequencies of pesticide application** | | | | |
| --- | --- | --- | --- | --- | --- |
|  | **In past 3 days** | **In past 3 days*winter** | **In past 4-10 days** | **In past 4-10 days*winter** | **Constant** |
| **Blood routine** |  |  |  |  |  |
| RBC | -0.20** | 0.11 | -0.09 | 0.14* | 4.68** |
|  | (0.06) | (0.09) | (0.05) | (0.06) | (0.03) |
| MCV | 2.29** | -1.67 | 1.12* | -0.92 | 91.77** |
|  | (0.70) | (0.98) | (0.53) | (0.73) | (0.31) |
| MCH | 0.79** | -0.18 | -0.13 | 0.06 | 30.96** |
|  | (0.27) | (0.39) | (0.21) | (0.29) | (0.12) |
| MCHC | 1.02 | 4.13 | -5.35** | 4.00* | 336.60** |
|  | (1.86) | (2.61) | (1.40) | (1.94) | (0.83) |
| RDW_CV | 0.06 | -0.43* | 0.07 | 0.05 | 12.57** |
|  | (0.12) | (0.17) | (0.09) | (0.13) | (0.05) |
| PLT | -18.54** | 17.99* | 1.83 | -7.26 | 215.70** |
|  | (5.82) | (8.19) | (4.39) | (6.07) | (2.60) |
| **Hepatic function** |  |  |  |  |  |
| ALT | 4.33** | 0.03 | 0.08 | -1.07 | 21.38** |
|  | (1.42) | (2.00) | (1.07) | (1.48) | (0.63) |
| AST | 4.71** | -2.16 | -1.27 | 0.49 | 24.04** |
|  | (1.37) | (1.93) | (1.03) | (1.43) | (0.61) |
| CHE | -355.20** | 66.86 | -327.80** | 195.20 | 8619.00** |
|  | (133.50) | (187.70) | (100.70) | (139.10) | (59.62) |
| **Renal function** |  |  |  |  |  |
| Cr | -3.72* | 4.64 | -0.50 | 0.10 | 75.23** |
|  | (1.79) | (2.52) | (1.35) | (1.87) | (0.80) |
| **Electrolytes** |  |  |  |  |  |
| Na | -0.89** | 0.37 | -0.57 | 0.39 | 141.80** |
|  | (0.38) | (0.54) | (0.29) | (0.40) | (0.17) |
| P | -0.01 | 0.15** | 0.00 | -0.05 | 1.24** |
|  | (0.03) | (0.05) | (0.03) | (0.04) | (0.02) |
| **Vitamins** |  |  |  |  |  |
| VB_12_ | 59.12 | -154.70** | 71.78* | -58.41 | 470.50** |
|  | (41.48) | (58.34) | (31.29) | (43.23) | (18.53) |
| **Glucose** |  |  |  |  |  |
| Glu | -0.33** | 0.28 | -0.29** | 0.29** | 5.31** |
|  | (0.11) | (0.15) | (0.08) | (0.11) | (0.05) |
| **Conduction velocity** |  |  |  |  |  |
| MNMCV | 0.63 | -0.15 | 0.55 | -1.34* | 59.14** |
|  | (0.52) | (0.67) | (0.41) | (0.54) | (0.23) |
| UNSCV | 1.10 | -0.36 | 0.28 | 0.00 | 53.47** |
|  | (0.62) | (0.80) | (0.49) | (0.64) | (0.28) |
| **Amplitude** |  |  |  |  |  |
| MNPCMAPA | -0.90* | 0.57 | -0.52 | 0.85 | 13.18** |
|  | (0.38) | (0.49) | (0.30) | (0.40) | (0.17) |
| MNDCMAPA | -0.96** | 0.83 | -0.34 | 0.82 | 13.66** |
|  | (0.37) | (0.48) | (0.29) | (0.39) | (0.17) |
| UNPCMAPA | -0.83** | 0.29 | -0.43* | 0.49 | 12.20** |
|  | (0.26) | (0.34) | (0.20) | (0.27) | (0.12) |
| UNDCMAPA | -0.62* | 0.42 | -0.42* | 0.50* | 12.83** |
|  | (0.24) | (0.31) | (0.19) | (0.25) | (0.11) |
| MNSNAPA | -0.82** | 0.21 | -0.01 | 0.35 | 8.16** |
|  | (0.25) | (0.32) | (0.20) | (0.26) | (0.11) |
| **MMSE** |  |  |  |  |  |
| MMSE | -0.36 | 0.75 | 0.81** | -0.89* | 26.04** |
|  | (0.36) | (0.48) | (0.27) | (0.37) | (0.16) |

** and * indicate the statistically significant at 1% and 5%, respectively.
