# Supplementary material for: Long- and Short-Term Health Effects of Pesticide Exposure: A Cohort Study from China
Source: PLoS One. 2015 Jun 4;10(6):e0128766. doi: 10.1371/journal.pone.0128766 (PMC4456378; doi:10.1371/journal.pone.0128766)
Supplement: S8 Table — (DOCX) [file pone.0128766.s008.docx]

**S8 Table. Estimated results of the short-term effects on blood routine adjusting for regions (FE estimation).**

| **Dependent variables:**  **∆Indicator** | **Independent variables: ∆ frequencies of pesticide application** | | | | | | **Constant** |
| --- | --- | --- | --- | --- | --- | --- | --- |
|  | **In past 3 days** | **In past 3 days*Hebei** | **In past 3 days*Guangdong** | **In past 4-10 days** | **In past 4-10 days*Hebei** | **In past 4-10 days*Guangdong** |  |
| WBC | -0.20 | 0.15 | 0.49 | 0.02 | 0.33 | -0.11 | 6.25** |
|  | (0.21) | (0.93) | (0.28) | (0.18) | (0.31) | (0.23) | (0.09) |
| Neu | -0.12 | -0.01 | 0.21 | -0.04 | 0.28 | -0.05 | 3.71** |
|  | (0.15) | (0.70) | (0.21) | (0.13) | (0.23) | (0.17) | (0.07) |
| Lym | -0.09 | 0.05 | 0.15 | -0.01 | 0.13 | 0.01 | 2.07** |
|  | (0.11) | (0.50) | (0.15) | (0.09) | (0.17) | (0.12) | (0.05) |
| Mon | -0.12** | 0.02 | 0.06 | -0.09** | -0.11* | 0.12* | 0.36** |
|  | (0.03) | (0.16) | (0.05) | (0.03) | (0.05) | (0.04) | (0.01) |
| Neup | -0.18 | -1.03 | -0.82 | -0.97 | 1.43 | 0.39 | 59.39** |
|  | (1.04) | (4.73) | (1.42) | (0.90) | (1.59) | (1.15) | (0.44) |
| Lymp | -0.64 | -0.04 | 0.02 | 0.45 | -0.19 | -0.04 | 33.01** |
|  | (0.95) | (4.31) | (1.30) | (0.82) | (1.45) | (1.05) | (0.40) |
| Monp | -2.22** | 0.94 | 1.16 | -1.62** | -1.88* | 2.16** | 5.88** |
|  | (0.55) | (2.52) | (0.76) | (0.48) | (0.85) | (0.61) | (0.23) |
| RBC | -0.20** | -0.13 | 0.11 | -0.09 | -0.01 | 0.13 | 4.68** |
|  | (0.06) | (0.29) | (0.09) | (0.06) | (0.10) | (0.07) | (0.03) |
| Hb | -1.82 | -5.00 | 2.26 | -4.60** | 4.16 | 5.96** | 143.60** |
|  | (1.41) | (6.42) | (1.93) | (1.23) | (2.16) | (1.56) | (0.60) |
| Hct | -0.64 | -0.70 | 0.12 | -0.84 | 1.60 | 1.40* | 42.56** |
|  | (0.59) | (2.67) | (0.80) | (0.51) | (0.90) | (0.65) | (0.25) |
| MCV | 2.78** | 0.84 | -2.16* | 0.08 | 3.11** | 0.13 | 91.70** |
|  | (0.72) | (3.29) | (0.99) | (0.63) | (1.11) | (0.80) | (0.31) |
| MCH | 0.95** | -0.47 | -0.33 | -0.41 | 0.85 | 0.34 | 30.94** |
|  | (0.29) | (1.31) | (0.40) | (0.25) | (0.44) | (0.32) | (0.12) |
| MCHC | 0.87 | -5.74 | 4.28 | -4.61** | -2.02 | 3.27 | 336.70** |
|  | (1.97) | (8.95) | (2.69) | (1.71) | (3.00) | (2.17) | (0.83) |
| RDW_CV | 0.05 | -0.03 | -0.43* | 0.07 | -0.01 | 0.04 | 12.57** |
|  | (0.13) | (0.58) | (0.18) | (0.11) | (0.20) | (0.14) | (0.05) |
| PLT | -17.78** | 17.09 | 17.23* | -1.00 | 7.94 | -4.43 | 215.50** |
|  | (6.17) | (28.05) | (8.44) | (5.36) | (9.42) | (6.81) | (2.61) |
| MPV | 0.27 | -0.88 | -0.79** | -0.05 | -0.44 | 0.22 | 10.62** |
|  | (0.18) | (0.82) | (0.25) | (0.16) | (0.27) | (0.20) | (0.08) |
| PDW | 0.26 | 1.05 | 0.68 | 0.64* | 0.40 | -0.88** | 14.71** |
|  | (0.30) | (1.36) | (0.41) | (0.26) | (0.46) | (0.33) | (0.13) |

** and * indicate the statistically significant at 1% and 5%, respectively.
