# Supplementary material for: Long- and Short-Term Health Effects of Pesticide Exposure: A Cohort Study from China
Source: PLoS One. 2015 Jun 4;10(6):e0128766. doi: 10.1371/journal.pone.0128766 (PMC4456378; doi:10.1371/journal.pone.0128766)
Supplement: S9 Table — (DOCX) [file pone.0128766.s009.docx]

**S9 Table. Estimated results of the short-term effects on blood biochemistry and C-reactive protein adjusting for regions (FE estimation).**

| **Dependent variables:**  **∆Indicator** | **Independent variables: ∆ frequencies of pesticide application** | | | | | | **Constant** |
| --- | --- | --- | --- | --- | --- | --- | --- |
|  | **In past 3 days** | **In past 3 days*Hebei** | **In past 3 days*Guangdong** | **In past 4-10 days** | **In past 4-10 days*Hebei** | **In past 4-10 days*Guangdong** |  |
| **Hepatic function** |  |  |  |  |  |  |  |
| ALT | 3.21* | 14.71* | 1.15 | 1.22 | -3.89 | -2.21 | 21.46** |
|  | (1.49) | (6.77) | (2.04) | (1.29) | (2.27) | (1.64) | (0.63) |
| AST | 3.18* | 8.05 | -0.63 | 1.21 | -7.69** | -1.99 | 24.22** |
|  | (1.42) | (6.44) | (1.94) | (1.23) | (2.16) | (1.56) | (0.60) |
| CHE | -328.80* | -199.10 | 43.41 | -360.00** | 103.10 | 227.40 | 8617.00** |
|  | (141.80) | (644.80) | (194.10) | (123.30) | (216.40) | (156.50) | (60.80) |
| TP | -1.90* | -2.70 | -2.41 | -1.87* | -1.18 | 2.74** | 74.38** |
|  | (0.96) | (4.35) | (1.31) | (0.83) | (1.46) | (1.06) | (0.41) |
| **Renal function** |  |  |  |  |  |  |  |
| Urea | 0.36* | -1.58* | -0.19 | -0.04 | 0.26 | 0.05 | 5.25** |
|  | (0.16) | (0.75) | (0.22) | (0.14) | (0.25) | (0.18) | (0.07) |
| Cr | -1.77 | -6.34 | 2.69 | -3.96* | 10.57** | 3.56 | 74.98** |
|  | (1.85) | (8.39) | (2.53) | (1.61) | (2.82) | (2.04) | (0.78) |
| **Electrolytes** |  |  |  |  |  |  |  |
| Na | -0.86* | 2.38 | 0.34 | -0.82* | 0.67 | 0.54 | 141.80** |
|  | (0.40) | (1.82) | (0.55) | (0.35) | (0.61) | (0.44) | (0.17) |
| K | 0.08 | -0.44 | -0.25* | -0.02 | -0.08 | 0.03 | 4.57** |
|  | (0.09) | (0.40) | (0.12) | (0.08) | (0.13) | (0.10) | (0.04) |
| P | -0.01 | 0.02 | 0.15** | -0.00 | 0.01 | -0.05 | 1.24** |
|  | (0.04) | (0.17) | (0.05) | (0.03) | (0.06) | (0.04) | (0.02) |
| **Vitamins** |  |  |  |  |  |  |  |
| VB_12_ | 56.84 | 291.30 | -152.40* | 54.28 | 42.93 | -40.90 | 469.20** |
|  | (43.74) | (198.90) | (59.88) | (38.03) | (66.77) | (48.27) | (18.54) |
| Folic acid | -0.38 | 4.15 | 0.97 | 0.04 | 2.96** | 0.09 | 9.09** |
|  | (0.70) | (3.16) | (0.95) | (0.60) | (1.06) | (0.77) | (0.29) |
| **Glucose** |  |  |  |  |  |  |  |
| Glu | -0.35** | 0.16 | 0.30 | -0.28** | -0.05 | 0.28 | 5.31** |
|  | (0.11) | (0.52) | (0.16) | (0.10) | (0.17) | (0.13) | (0.05) |
| **C-reactive protein** |  |  |  |  |  |  |  |
| CRP | 0.35 | 0.82 | -0.69 | -0.78 | -0.10 | 0.91 | 1.55** |
|  | (0.64) | (2.91) | (0.88) | (0.56) | (0.98) | (0.71) | (0.27) |

** and * indicate the statistically significant at 1% and 5%, respectively.
