# Supplementary material for: Long- and Short-Term Health Effects of Pesticide Exposure: A Cohort Study from China
Source: PLoS One. 2015 Jun 4;10(6):e0128766. doi: 10.1371/journal.pone.0128766 (PMC4456378; doi:10.1371/journal.pone.0128766)
Supplement: S10 Table — (DOCX) [file pone.0128766.s010.docx]

**S10 Table. Estimated results of the short-term effects on farmer’s nerve conduction studies and neurological examination adjusting for regions (FE estimation).**

| **Dependent variables:**  **∆Indicator** | **Independent variables: ∆ frequencies of pesticide application** | | | | | | **Constant** |
| --- | --- | --- | --- | --- | --- | --- | --- |
|  | **In past 3 days** | **In past 3 days *Hebei** | **In past 3 days *Guangdong** | **In past 4-10 days** | **In past 4-10 days *Hebei** | **In past 4-10 days *Guangdong** |  |
| **Conduction velocity** |  |  |  |  |  |  |  |
| MNMCV | 0.28 | -2.22 | 0.21 | 1.24* | -1.75* | -2.03** | 59.18** |
|  | (0.56) | (2.48) | (0.70) | (0.52) | (0.84) | (0.62) | (0.23) |
| UNMCV | 1.01 | -0.08 | -0.74 | 0.31 | -0.17 | -0.14 | 57.21** |
|  | (0.53) | (2.34) | (0.66) | (0.49) | (0.79) | (0.59) | (0.22) |
| TNMCV | -0.45 | 0.47 | 0.47 | -0.02 | -0.65 | -0.43 | 47.93** |
|  | (0.48) | (2.15) | (0.61) | (0.45) | (0.73) | (0.54) | (0.20) |
| PNMCV | -0.49 | -1.98 | 0.87 | 0.46 | -0.64 | -0.32 | 48.46** |
|  | (0.38) | (1.70) | (0.48) | (0.35) | (0.57) | (0.43) | (0.16) |
| MNSCV | 0.61 | -1.27 | -0.92 | 1.52** | -2.76 | -1.28 | 58.01** |
|  | (0.59) | (2.62) | (0.74) | (0.55) | (0.88) | (0.66) | (0.25) |
| UNSCV | 0.67 | -2.43 | 0.07 | 1.26* | -2.45* | -0.98 | 53.52** |
|  | (0.66) | (2.93) | (0.83) | (0.61) | (0.99) | (0.74) | (0.28) |
| SNSCV | 0.13 | -1.60 | 0.43 | -0.09 | -1.37 | 0.31 | 59.93** |
|  | (0.72) | (3.21) | (0.90) | (0.67) | (1.08) | (0.81) | (0.30) |
| **Distal motor latency** |  |  |  |  |  |  |  |
| MNDML | -0.08 | 0.13 | 0.04 | -0.06 | 0.04 | -0.03 | 3.46** |
|  | (0.08) | (0.36) | (0.10) | (0.08) | (0.12) | (0.09) | (0.03) |
| UNDML | -0.05 | 0.11 | -0.15 | -0.18* | 0.16 | 0.18 | 2.81** |
|  | (0.08) | (0.37) | (0.10) | (0.08) | (0.13) | (0.09) | (0.03) |
| TNDML | 0.01 | -0.04 | -0.12 | -0.01 | 0.08 | 0.11 | 3.66** |
|  | (0.06) | (0.29) | (0.08) | (0.06) | (0.10) | (0.07) | (0.03) |
| PNDML | 0.09 | 0.03 | -0.18* | -0.08 | 0.11 | 0.09 | 3.73** |
|  | (0.07) | (0.32) | (0.09) | (0.07) | (0.11) | (0.08) | (0.03) |
| **Amplitude** |  |  |  |  |  |  |  |
| MNPCMAPA | -1.24** | -0.55 | 0.91 | 0.16 | -1.72** | 0.17 | 13.22** |
|  | (0.41) | (1.80) | (0.51) | (0.38) | (0.61) | (0.45) | (0.17) |
| MNDCMAPA | -1.17** | -1.03 | 1.04* | 0.14 | -1.19* | 0.35 | 13.69** |
|  | (0.40) | (1.78) | (0.50) | (0.37) | (0.60) | (0.45) | (0.17) |
| UNPCMAPA | -0.80** | 0.99 | 0.26 | -0.56* | 0.30 | 0.62* | 12.19** |
|  | (0.28) | (1.25) | (0.35) | (0.26) | (0.42) | (0.31) | (0.12) |
| UNDCMAPA | -0.57* | 1.67 | 0.38 | -0.59* | 0.40 | 0.67* | 12.82** |
|  | (0.26) | (1.14) | (0.32) | (0.24) | (0.39) | (0.29) | (0.11) |
| TNPCMAPA | 0.31 | 0.62 | -0.53 | -0.60* | 1.05* | 0.20 | 11.35** |
|  | (0.32) | (1.41) | (0.40) | (0.29) | (0.48) | (0.36) | (0.13) |
| TNDCMAPA | 0.89* | 1.04 | -1.03* | -0.37 | 1.16 | 0.36 | 13.77** |
|  | (0.41) | (1.80) | (0.51) | (0.38) | (0.61) | (0.45) | (0.17) |
| PNPCMAPA | -0.40 | 0.06 | 0.43 | -0.05 | -0.47 | -0.10 | 6.78** |
|  | (0.24) | (1.05) | (0.30) | (0.22) | (0.35) | (0.26) | (0.10) |
| PNDCMAPA | -0.52* | -0.16 | 0.80** | 0.01 | 0.06 | -0.27 | 7.37** |
|  | (0.25) | (1.09) | (0.31) | (0.23) | (0.37) | (0.27) | (0.10) |
| MNSNAPA | -0.65* | -1.31 | 0.04 | -0.24 | 0.63 | 0.58 | 8.15** |
|  | (0.27) | (1.19) | (0.34) | (0.25) | (0.40) | (0.30) | (0.11) |
| UNSNAPA | -0.32 | -1.24 | -0.03 | 0.02 | -0.04 | -0.15 | 6.20** |
|  | (0.21) | (0.94) | (0.27) | (0.20) | (0.32) | (0.24) | (0.09) |
| SNSNAPA | -0.23 | 0.93 | -0.07 | -0.90 | 1.34 | 1.40 | 15.79** |
|  | (0.73) | (3.23) | (0.91) | (0.67) | (1.09) | (0.81) | (0.30) |
| **TNSc** |  |  |  |  |  |  |  |
| TNSc | 0.32 | 3.13* | -0.13 | 0.16 | 0.88 | -0.33 | 2.57** |
|  | (0.30) | (1.38) | (0.40) | (0.26) | (0.46) | (0.33) | (0.13) |
| **MMSE** |  |  |  |  |  |  |  |
| MMSE | -0.47 | 3.46* | 0.86 | 0.79* | -0.02 | -0.87 | 26.04** |
|  | (0.37) | (1.71) | (0.49) | (0.33) | (0.57) | (0.41) | (0.16) |

** and * indicate the statistically significant at 1% and 5%, respectively.
